# Effect of β-alanine on the athletic performance and blood amino acid metabolism of speed-racing Yili horses

**DOI:** 10.3389/fvets.2024.1339940

**Published:** 2024-02-28

**Authors:** Xiaobin Li, Jun Ma, Haiying Li, Hai Li, Yuhui Ma, Haifeng Deng, Kailun Yang

**Affiliations:** ^1^Xinjiang Key Laboratory of Herbivore Nutrition for Meat and Milk Production, College of Animal Science, Xinjiang Agricultural University, Ürümqi, China; ^2^Xinjiang Key Laboratory of Horse Breeding and Exercise Physiology, College of Animal Science, Xinjiang Agricultural University, Ürümqi, China; ^3^General Animal Husbandry Station of Ili Kazakh Autonomous Prefecture, Yining, China

**Keywords:** β-alanine, carnosine, Yili horse, free amino acids, athletic performance

## Abstract

The objective of this study was to examine the effects of supplemental β-alanine feeding on the athletic performance of Yili horses involved in speed racing, focusing on alterations in plasma free amino acid patterns pre and post exercise. Additionally, the research aimed to evaluate the effects of carnosine on the plasma acid–base buffering capacity and antioxidant levels in these horses. Twelve Yili horse stallions, averaging 3 years in age and 346.50 ± 21.39 kg in weight, were chosen and randomly divided into two groups: a control group and a test group, each comprising six horses. The control group received a supplementation of 300 mg/kg BW/day of α-alanine, while the test group received 300 mg/kg BW/day of β-alanine. This supplementation regimen was maintained for a 30-day supplementation trial period, under identical feeding and management conditions. Throughout the trial, the horses participated in the 1,000 Speed Race, and three distinct blood samples were gathered for assessing plasma free amino acids, blood gases, biochemical parameters, and antioxidant parameters. The outcomes indicated a considerable enhancement in the 1,000 m exercise performance of the speed racing Yili horses in the test group compared to the control group, showcasing a noteworthy improvement of 12.01%, with the test group completing the race 13.29 s faster. Notably, the α-alanine content in the plasma of the control group Yili horses remained higher than that of the test group, demonstrating a consistent increasing trend. By contrast, the plasma β-alanine content was notably higher in the test group than in the control group. Over the course of the supplementation period, plasma β-alanine exhibited an escalating and then stabilizing trend in the test group, whereas in the control group, although β-alanine content also increased, the trend was less pronounced. The plasma levels of histidine and carnosine showed minimal variance between the two groups. Overall, the test group of Yili horses exhibited slightly higher plasma levels of histidine and carnosine compared to the control group. The addition of β-alanine to their diet for a duration of 30 days notably affected the plasma levels of amino acids both pre- and post-exercise in speed-racing Yili horses. Furthermore, β-alanine demonstrated an inhibitory effect on the catabolism of these horses’ bodies during high-intensity exercise. Ten marker amino acids, including valine, leucine, β-alanine, isoleucine, carnosine, 3-methyl-histidine, lysine, ethanolamine, argnine, and taurine, displayed statistically significant changes. β-alanine notably increased the blood glucose levels of Yili horses and played a role in expediting the restoration of blood gas levels post-exercise. Moreover, in the test group of Yili horses, the levels of superoxide dismutase, glutathione peroxidase, and total antioxidant capacity significantly increased both before and after the race, while the content of malondialdehyde, an oxidation product, exhibited an extremely significant decrease immediately after the race. These outcomes suggest that the addition of β-alanine significantly augmented antioxidant levels during high-intensity exercise in Yili horses. Consequently, it reduced post-exercise injuries and accelerated the recovery process after exercise.

## 1 Introduction

Carnosine, an endogenous water-soluble dipeptide, was discovered in 1900 by Guiotto and others. It is widely present in mammalian skeletal muscle, brain, nerves, and various tissues (1–3). The synthesis of carnosine in skeletal muscle is catalyzed by myostatin synthetase, which begins with the pre-activation of β-alanine under ATP-energy conditions to produce the “enzyme-β-alanyl adenosine” complex, followed by the binding of the β-alanyl portion of the enzyme to L-histidine, and the final release of carnosine from the enzyme (4). Carnosine demonstrates strong amphoteric electrolyte characteristics and effective acid–base buffering capacity (5, 6). The molecular structure of carnosine, particularly the imidazole ring and amino residue, grants it robust antioxidant properties (7, 8). In research by Huang et al., β-alanine supplementation was found to increase myostatin content in skeletal muscle. Myostatin, primarily located in myocytes, contributes to maintaining myocytes’ internal stability, regulating physiological pH, chelating metal ions, scavenging free radicals, and managing glucose metabolism during intense physical activity. Elevated myostatin levels in skeletal muscle can enhance exercise performance, owing to its exceptional antioxidant properties that shield mitochondria from oxidative damage caused by free radicals, ensuring a continuous energy supply (9). In summary, β-alanine can improve mitochondrial damage, promote mitochondrial integrity, improve energy metabolism and the body’s ability to resist oxidative stress during exercise and thus promote exercise performance. Thus, increasing the body’s carnosine levels improves the body’s exercise capacity (10, 11). Despite its physiological significance, carnosine is inadequately present and utilized in food or feed. Studies indicate that supplementing with β-alanine, which is deficient in common dietary sources, significantly elevates carnosine levels in the body (12–14). The Yili Horse 1,000 m Speed Race is a high-intensity event that requires a high level of energy supply and resistance to exercise stress. Therefore, in the present study, we supplemented β-alanine to 1,000 m speed horses, and investigated whether the level of 1,000 m speed race and the ability of resistance to exercise stress could be improved by increasing the level of myostatin in the body of the horses through detecting the changes in the exercise level of the horses, the concentration of amino acids in the blood, and the indexes of antioxidant and blood gases before and after the race.

## 2 Materials and methods

### 2.1 Ethical considerations

This study protocol received approval from the Animal Care and Utilization Committee of the College of Animal Science, Xinjiang Agricultural University (Urumqi, China). The research was conducted at Zhaosu Horse Farm in Yili Kazakh Autonomous Prefecture, Xinjiang.

### 2.2 Animal and experimental design

Twelve Yili horse stallions, aged 3 years and weighing an average of (346.50 ± 21.39 kg), with similar 1,000-m speed race performances (135.24 ± 15.75 s) from Zhaosu stable, were selected for this study. They were randomly divided into two groups of six stallions each: the control group and the test group. In accordance with Dunnet (15), the supplemental feeding dose was designed as 300 mg/kg BW/day α-alanine for the control group and 300 mg/kg BW/day β-alanine for the test group. Throughout the test period, each horse received a daily diet comprising 4 kg of high-quality mountain grass and 4 kg of alfalfa hay, along with a concentrate supplement of 3 kg in three divided doses per day, and had free access to water. Table 1 displays the diet composition and nutrient levels. The test horses were individually housed in single stalls, and their bedding was cleaned and replaced every 2 days. Feeding occurred at 07:00, 10:00, 13:00, 16:00, and 19:00 daily. Before feeding concentrate, a small amount of roughage was provided appropriately, and after finishing the roughage, the horse was allowed to move freely in the outdoor activity field. Initially, supplementation was approximately 10 g per horse, mixed with the concentrate supplement to prevent anorexia caused by the additive. The dose was gradually increased to normal levels. Table 2 outlines the training program for Yili horses in speed racing. The test horses underwent speed training once a day, scheduled during 10:00–12:00 or 14:00–16:00. The training involved the rider leading the horse to the training ground, commencing with a warm-up where the jockey slowly walked the test horse in the grass in a circular motion, then transitioned to the sand and gradually increased speed, followed by two laps of warm-up training before starting the formal speed training. Formal training utilized the interval training method, with intervals lasting 1–2 min. The volume of formal training remained constant throughout the trial period, while the training intensity gradually increased. The training intensity was gauged by the speed horses’ 1,000-m performance, with the rider controlling the horses’ speed. The intensity was progressively reduced from 1.25 times the time taken to complete the 1,000 m at maximum speed in the initial stages of training to 1.11 times in the later stages.

**Table 1:** Diet composition and nutrient levels (DM basis).

Items	Content (%)
**Ingredients %**	
Alfalfa hay	72.73
Corn	15.00
Bran	3.27
Soybean meal	7.36
CaHPO_4_	0.82
NaCl	0.55
Premixes^1^	0.27
Total	100
**Nutrient levels** ^**2**^	
DM (%)	92.10
OM (%)	92.56
CP (%)	12.92
NDF (%)	20.76
ADF (%)	33.12
Ca (%)	0.86
P (%)	0.09
DM (%)	92.10

^1^The premix provided the following per kg of diets: VA 4.09 mg, VB1 5.80 mg, VB2 2.84 mg, VB6 0.33. Mg, VB12 0.0027 mg, VC 5.68 mg, VD 0.60 mg, VE 218.12 mg. Calcium Pantothenate 1.39 mg, Nicotinamide 3.31 mg, Biotin 2.73 mg, Choline chloride 32.73 mg, Swell soil 1.94 g, Cu (as copper sulfate) 45.09 mg, Fe (as ferrous sulfate) 151.52 mg, Mn (as manganese sulfate) 142.94 mg, Zn (as zinc sulfate) 131.75 mg, I (as potassium iodide) 10.53 mg, Se (as sodium selenite) 25.25 mg and Co (as cobalt chloride) 4.55 mg. ^2^The nutrient levels are all measured values.

**Table 2:** Training program for speed racing Yili horses.

Train time(d)	Warm-up before treatment	Training intensity	Distance	Intermissive time	Repetitional times
1–14 days	The test horses were warmed up first at a slow trot on the grass track and the sand track respectively, and gradually increased their speed, and after two warm-up laps they officially started interval training	1.25 times the time of the 1,000 m speed event	1,000 m	1–2 min	3
15–30 days		1.11 times the time of the 1,000 m speed event	1,000 m	1–2 min	3

### 2.3 Sample collection and analysis

A 1,000-m speed simulation test race took place at the Westfield Racecourse on the day before the commencement of the trial, on the 15th day, and on the 30th day. Professional riders were invited to participate in the race. The event involved four start/finish judges, four timekeepers, and one data recorder. During the race, the timekeepers were responsible for accurately determining the test horses’ simulated race score. The average of the valid data was considered as the horses’ test race score after validation by the judges, and the scores from the three simulated test race competitions were recorded.

In this trial, three types of blood samples were collected to determine plasma free amino acids, blood gases, biochemical indexes, and antioxidant indexes. To determine plasma free amino acids, blood samples were collected from the horses’ jugular veins using EDTA anticoagulant tubes. These samples, measuring 5 mL each, were collected 1 h before, immediately after, and 2 h after the simulated test match on the 30th day of the trial. The collected blood samples were centrifuged at 1,500 × g for 15 min immediately after collection, and the resulting plasma was stored at −80°C.

Blood samples for measuring blood gases and biochemical analyses were obtained from the jugular vein of the horses using regular blood collection tubes at a rate of 3 mL per test. For analysis, 100 μL of whole blood, not exposed to air below the liquid level, was aspirated. Sampling times were 1 h before, immediately after, 20 min after, and 2 h after the simulated test match on the 30th day of the trial period.

Blood gas parameters were measured immediately after the collection of blood samples. For the determination of antioxidant indices, blood samples were collected in sodium heparin anticoagulant tubes from the jugular vein of the horses. These samples, measuring 5 mL each, were collected 1 h before the simulated test match on the 30th day of the trial and immediately after, 20 min after, and 2 h after the match. Similar to the previous samples, these were centrifuged at 1,500 × *g* for 15 min immediately after collection, and the resulting plasma was stored at −80°C.

### 2.4 Sample determination

#### 2.4.1 Determination of β-alanine-based indicators in plasma

Plasma β-alanines were analyzed by Beijing Purui Huasheng Biological Co., Ltd., using high-performance liquid chromatography–tandem mass spectrometry (HPLC-MS/MS).

##### 2.4.1.1 Sample processing

For the analysis, 50 μL of plasma was mixed with 50 μL of protein precipitant (containing NVL), frozen, and centrifuged at 13,200 rpm for 4 min. Then, 10 μL of the supernatant was mixed with 50 μL of labeling buffer, followed by the addition of 20 μL of derivatization solution. This mixture was subjected to constant temperature derivatization at 55°C for 15 min. After cooling in the refrigerator, the samples were dissociated, and 50 μL of the samples were used for testing in the machine.

##### 2.4.1.2 Instrument model

HPLC-MS/MS (Ultimate3000-API 3200 Q TRAP).

##### 2.4.1.3 Amino acid kit

MSLAB-45 + AA (batch no.: MSLAB451561#; Beijing Mass Spectrometry Medical Research Co.), methanol, and hadronitrile were purchased from Fisher.

The chromatographic conditions were as follows: column, MSLab 45 + AA − C18 (150 × 4.6 mm, 5 μm); mobile phase A, 0.1% formic acid aqueous solution; mobile phase B, acetonitrile; flow rate, 1 mL/min; column temperature, 50°C; injection volume, 5 μL; chromatographic run time, 20 min. The mass spectrometry conditions were as follows: the ion source was an +ESI electrospray ion source; MRM multi-reaction monitoring was used; the curtain gas (CUR) was 20 psi; spray voltage (ESI) was +5,500 V; collision gas (CAD) was medium; collision chamber ejection voltage (CXP) was 2.0; nebulizer (GAS1) was 55 psi; auxiliary gas (GAS2) was 60 psi; nebulization temperature (TEM) is 500°C; and ingress voltage was 10 V. Amino acid standard curves and peak times are shown in Table 3.

**Table 3:** Amino acid standard curve and peak time.

Item	Molecular weight	Peak time (minutes)	Standard curve	R^2^
3-Aib	103.12	7.38	y = 0.0126x + 0.00251	0.9994
3-MHis	169.18	3.78	y = 0.000406x − 3.12e − 005	0.9983
5Ava	117.15	7.93	y = 0.0189x + 0.000875	0.9997
5-HT	176.20	10.70	y = 0.0154x − 0.0021	0.9997
5-HTP	220.22	9.31	y = 0.00294x − 0.000907	0.9969
Aad	161.16	6.40	y = 0.00389x + 0.00708	0.9996
Abu	103.12	8.47	y = 0.0145x + 0.000688	0.9986
Ala	89.09	6.51	y = 0.00567x + 0.022	0.9965
Ans	240.26	3.82	y = 0.000176x − 3.58e − 005	0.9941
Arg	174.20	5.31	y = 0.00113x − 0.000548	0.9938
Asa	290.27	3.79	y = 0.00018x + 3.42e − 005	0.9932
Asn	132.12	3.26	y = 0.0101x + 0.00632	0.9974
Asp	133.10	4.63	y = 0.00221x + 0.000114	0.9949
β-Ala	89.09	5.64	y = 0.0108x + 0.023	0.9997
Car	226.23	3.80	y = 0.0108x + 0.023	0.9995
Cit	175.19	4.41	y = 0.000445x + 4.32e − 005	0.9974
Cth	222.26	7.95	y = 0.000603x + 0.000133	0.9997
Cys1	121.16	8.56	y = 0.000178x − 4.94e − 005	0.9974
EtN	61.08	4.56	y = 0.00188x-0.000229	0.9956
GABA	103.12	6.44	y = 0.0107x + 0.00522	0.9966
Gln	146.15	3.55	y = 0.00314x + 0.00217	0.9998
Glu	147.13	4.95	y = 0.00465x + 0.00118	0.9991
Gly	75.07	4.69	y = 0.00285x + 0.000531	0.9969
Harg	188.20	6.26	y = 0.000947x-6.12e − 005	0.9845
Hcit	189.20	5.64	y = 0.00107x + 0.000149	0.9988
His	155.15	3.76	y = 0.000377x − 9.31e − 005	0.9993
Hpro	129.16	10.00	y = 0.00436x − 0.000308	0.9990
Hser	119.10	3.73	y = 0.00276x + 0.00128	0.9917
Hyl	162.20	7.12	y = 0.00106x + 0.000221	0.9993
Hyp	131.13	3.12	y = 0.0274x + 0.00918	0.9927
Ile	131.18	12.70	y = 0.0211x + 0.000765	0.9995
Kyn	208.10	12.60	y = 0.00463x − 0.000324	0.9982
Leu	131.17	13.30	y = 0.0198x + 0.00132	0.9994
Lys	146.19	9.23	y = 0.00126x + 0.000487	0.9984
Met	149.20	10.10	y = 0.00965x + 0.000992	0.9996
Orn	132.16	8.32	y = 0.00133x + 0.000433	0.9991
PEtN	141.06	1.90	y = 0.000234x + 3.93e − 005	0.9973
Phe	165.19	13.9	y = 0.0174x + 0.00579	0.9988
Pro	115.14	7.08	y = 0.0109x + 0.0174	0.9997
Sar	89.09	5.21	y = 0.0118x + 0.00113	0.9997
Ser	105.09	3.74	y = 0.00761x + 0.0235	0.9908
Tau	125.25	2.02	y = 0.000735x-0.000721	0.9938
Thr	119.13	5.44	y = 0.0056x + 0.00018	0.9999
Trp	204.23	14.5	y = 0.0129x + 0.000269	0.9990
Tyr	181.19	9.47	y = 0.00799x + 0.00123	0.9989
Val	117.15	10.30	y = 0.0102x + 0.0145	0.9966

#### 2.4.2 Measurement of blood gases and biochemical indicators

Blood gas analysis is a crucial method to rapidly and accurately assess changes and trends in the body’s acid–base balance. It involves the determination of O_2_ and CO_2_ levels in the blood for gas exchange, along with measuring relevant acid–base balance parameters. This analysis aids in understanding the body’s ventilation, gas exchange function, and various acid–base imbalance conditions. In domestic animals, blood gas analysis helps comprehend the internal environment’s state concerning gas exchange, acid–base balance, and oxygenation. It is closely associated with electrolytes, essential for maintaining osmotic pressure and regulating acid–base balance and metabolism, including cardiac and muscle function regulation. The blood gas and biochemical indices were measured using an i-SATA portable blood gas analyzer. Electrochemical methods were used to determine Na^+^ and K^+^ levels, galvanometry for Glu and PO_2_, voltammetry for pH and PCO_2_, selective electrode voltammetry for iCa^2+^, and electroconductivity for Hct.

#### 2.4.3 Measurement of plasma antioxidant capacity

Plasma levels of malondialdehyde (MDA), total antioxidant capacity (T-AOC), glutathione peroxidase (GSH-px), superoxide dismutase (SOD), and catalase (CAT) activities were assessed using commercially available kits (Nanjing Jianjian Bioengineering Research Institute, Nanjing, China). The antioxidant enzyme activities were determined according to the provided kit manual. Measurements were conducted colorimetrically using a 7230G spectrophotometer for T-AOC, SOD, GSH-Px, CAT, and MDA in plasma.

### 2.5 Statistical analysis

Data of free amino acids and blood gas/biochemical indices were extracted and normalized using EZinfo2 2.0 software. Free amino acids PCA analyses were performed using the FactoMineR package in R software and the ggplot2 package (version 2.15.3). The PCoA analysis was performed using the WGCNA package, stat packages, and ggplot2 package in R software (Version 2.15.3).

Preliminary analysis of the experimental data was conducted using Excel 2010. The normality (Shapiro–Wilk) test was performed before statistical analysis. The Shapiro–Wilk test determined normality for athletic performance and plasma antioxidant indexes. Further statistical analysis was conducted using SPSS 20.0 software (SPSS Statistics 20, IBM Japan, Ltd., Tokyo, Japan) with independent-samples t-tests. Data were expressed as mean ± standard deviation, with *p* < 0.05 indicating significant differences and 0.05 < *p* < 0.10 indicating a significant trend of differences.

## 3 Results

### 3.1 Effect of supplemental feeding of β-alanine on the athletic performance of speed-racing Yili horses

**Figure 1:**
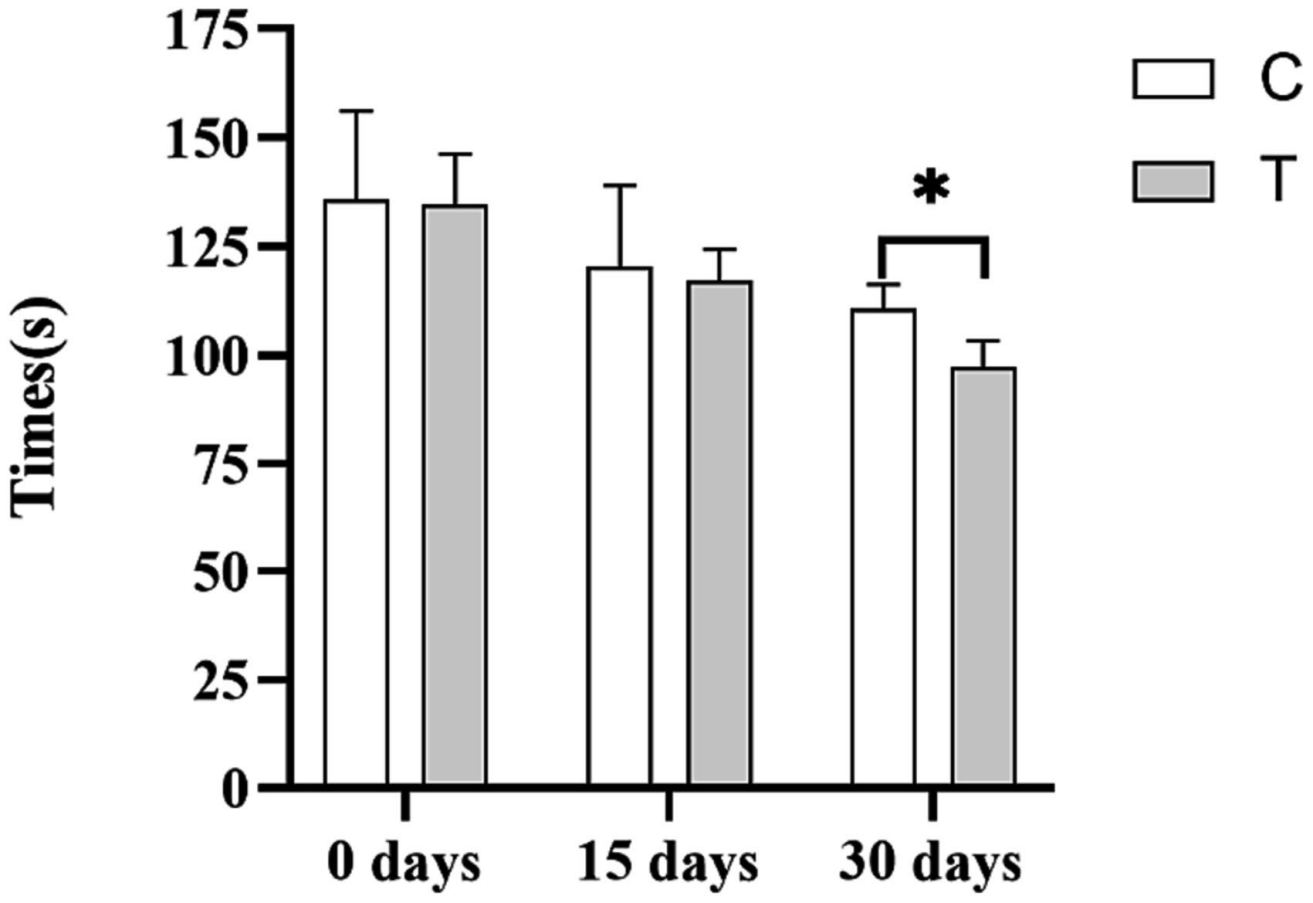
Effect of supplemental feeding of β-alanine on the athletic performance of speed-racing Yili horses (C: α-alanine group; T: β-alanine group). Each bar represents the mean of 6 horses ± standard error of the mean (SEM). * indicates a significant difference between the two groups (*p* < 0.05), ** indicates a highly significant difference between the two groups (*p* < 0.01).

Figure 1 depicts the effect of β-alanine supplementation on the athletic performance of Yili horses in speed racing. Initially, the 1,000-m exercise times for the control and test groups of speed racing Yili horses were 135.76 and 134.73 s, respectively. After 15 days, the performance of the test group improved by 2.47%, clocking in 2.97 s faster than the control group, although this difference was not statistically significant (*p* > 0.05). By day 30, the test group’s performance significantly improved by 12.01%, completing the 1,000-m race in 13.29 s less than that in the control group (*p* < 0.05).

### 3.2 Effect of supplemental feeding of β-alanine on β-alanine-like indices in the plasma of speed-racing Yili horses

**Figure 2:**
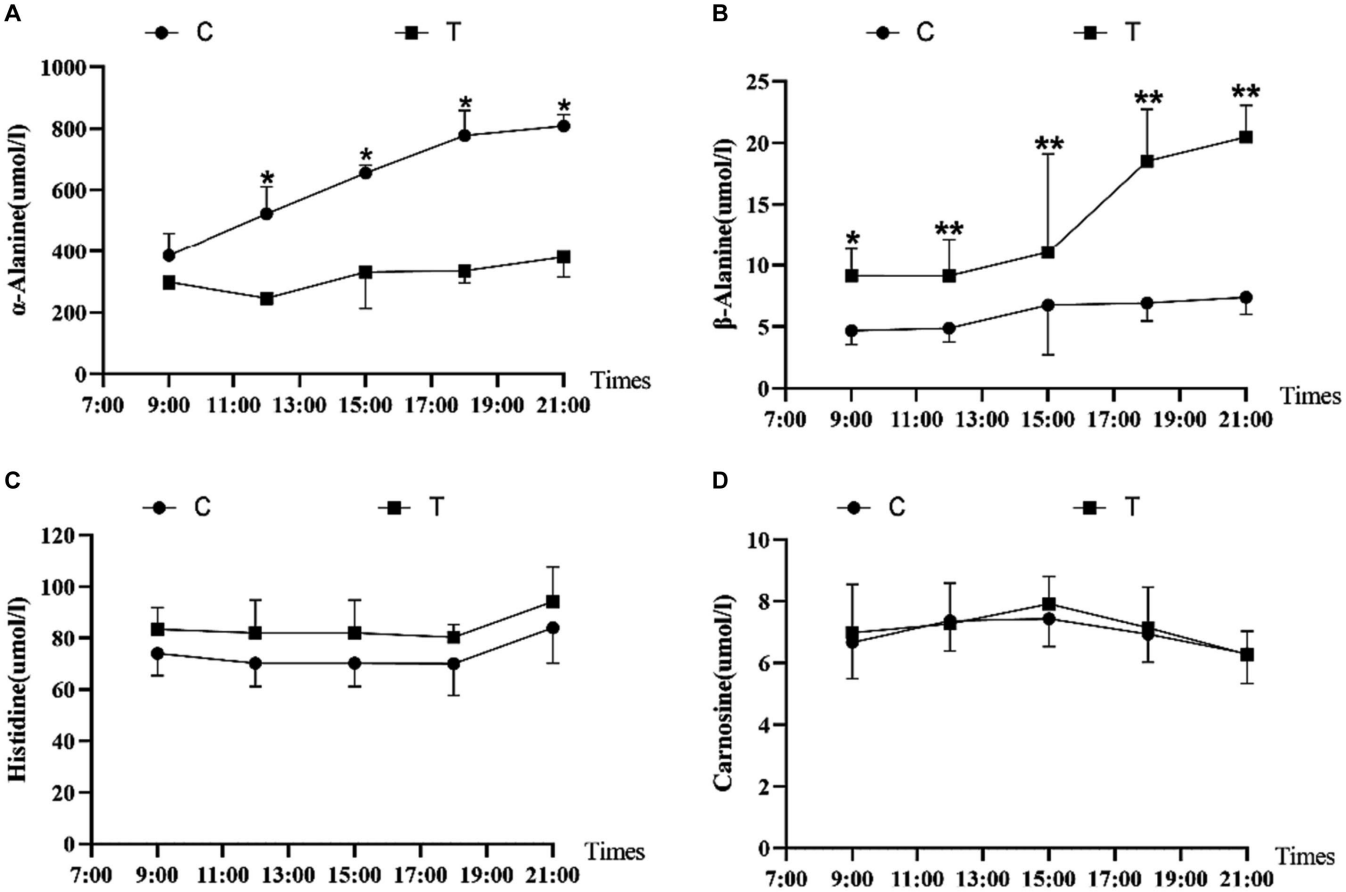
Effect of supplemental feeding of β-alanine on beta-alanine-like indices in the plasma of speed-racing Yili horses (**A–D**) (C: α-alanine group; T: β-alanine group). * indicates a significant difference between the two groups (*p* < 0.05), ** indicates a highly significant difference between the two groups (*p* < 0.01).

Figure 2 shows the effect of β-alanine supplementation on β-alanine-related parameters in the plasma of Yili horses during 1,000-m speed races. In Figure 2A, the plasma α-alanine content of the control group exhibited a steady increase, with a slower rising trend after 18:00 (i.e., the 4th supplemental feeding). Conversely, Figure 2B shows higher β-alanine content in the plasma of the test group than in the control group. The plasma β-alanine content remained stable from 9:00 to 15:00 (i.e., 2 h after the initial three supplemental feedings) and displayed a noticeable upward trend from 15:00 onward, with the most significant increase observed from 15:00 to 18:00 (i.e., 2 h after the third and fourth supplemental feedings). The upward trend had slowed down, and the concentration of α-alanine also increased, but the trend was not obvious. The control group also displayed an increase, but the trend was not obvious. Figures 2C,D indicate marginal differences in the plasma levels of histidine and carnosine between the test and control groups. Overall, the test group of Yili horses exhibited slightly higher plasma levels of histidine and carnosine than the control group.

### 3.3 Effect of supplemental feeding of β-alanine on amino acid metabolism in the plasma of speed-racing Yili horses

**Figure 3:**
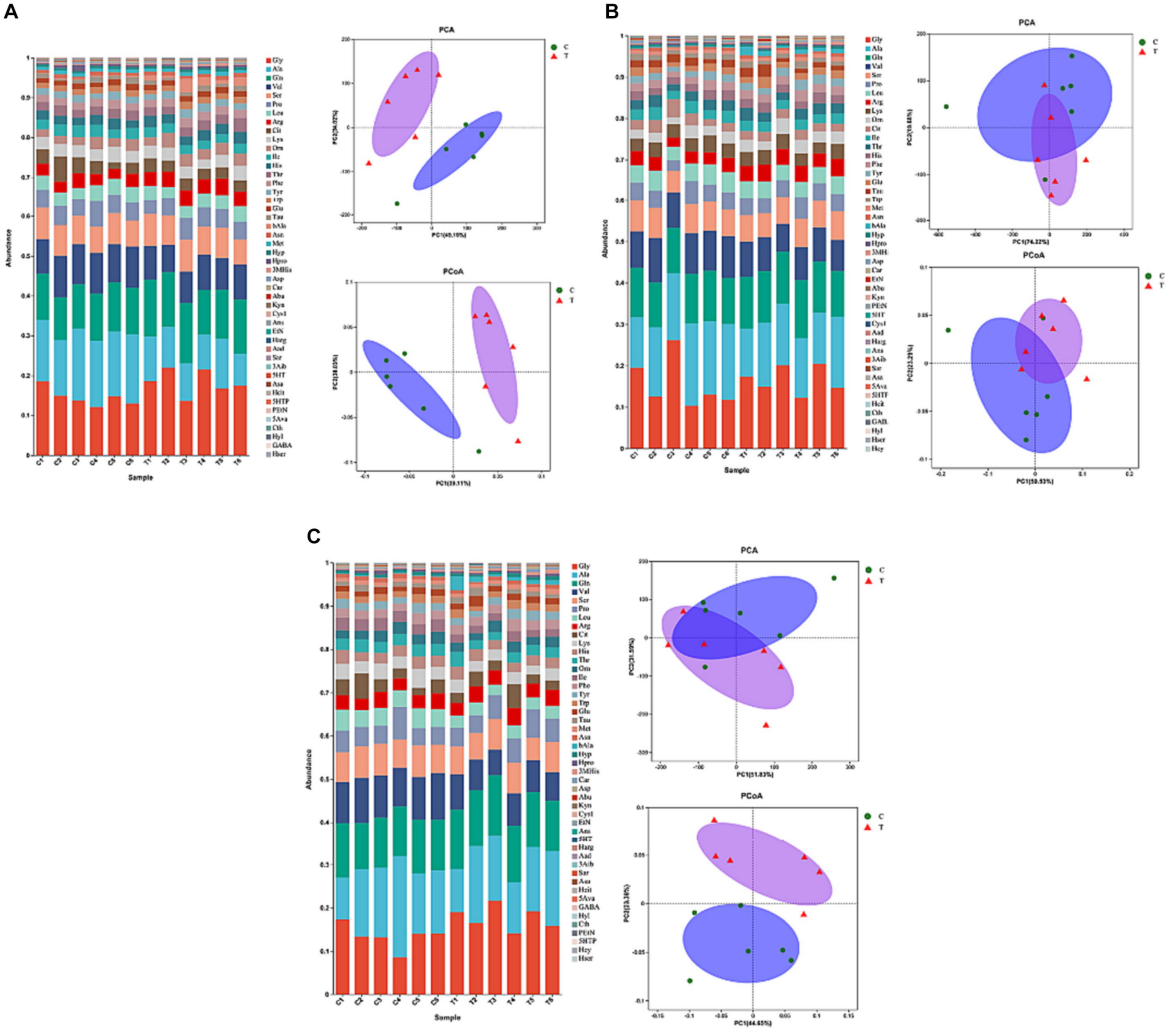
Effect of supplemental feeding of beta-alanine on amino acid metabolism in the plasma of speed-racing Yili horses (**A–C**) (C: α-alanine group T: β-alanine group).

Figure 3 illustrates the effect of β-alanine supplementation on amino acid metabolism in plasma both before and after speed racing among Yili horses. In Figure 3A and Table 4, the plasma concentration of β-alanine in the Yili horse test group was 10.09 times higher than that of the control group 1 h before the race (*p* < 0.01). Additionally, the concentration of valine was notably 14.67% lower than that of the control group (*p* < 0.05), while the concentration of arginine was significantly higher by 32.10% (*p* < 0.01). No other significant differences in amino acid concentrations were observed between the two groups. The principal coordinate analysis (PCoA) results demonstrated that the horizontal coordinate accounted for 39.11% of sample variation, with the vertical coordinate contributing 30.03%. Notably, there was no substantial similarity observed in amino acid concentrations within the pre-race plasma of control and test Yili horses based on the PCoA and principal component analysis (PCA). The PCA indicated that the horizontal coordinate, contributing 49.18% to sample variance, and the vertical coordinate, contributing 34.02%, did not reveal significant similarities in amino acid concentrations between the pre-race plasmas of control and test Yili horses.

**Table 4:** Effect of β-alanine on the metabolic concentration of amino acids in the plasma of Yili horses 1 h before the race (μmol/L).

Items	Control groups	Test groups
Gly	345.00 ± 95.92	441.67 ± 82.43
Ala	378.50 ± 38.68^A^	244.67 ± 63.75^B^
Gln	270.67 ± 30.07	324.00 ± 55.97
Val	229.50 ± 18.45^a^	195.83 ± 29.03^b^
Ser	172.00 ± 31.70	179.83 ± 33.89
Pro	114.62 ± 20.08	109.00 ± 11.47
Leu	87.63 ± 10.49	84.60 ± 16.22
Arg	70.88 ± 12.21^B^	93.63 ± 11.21^A^
Cit	85.70 ± 41.38	71.28 ± 15.53
Lys	74.33 ± 10.24	74.38 ± 14.71
Orn	54.62 ± 9.89^b^	70.07 ± 10.49^a^
Ile	62.93 ± 9.58	56.22 ± 9.30
His	53.32 ± 9.52	63.07 ± 11.97
Thr	54.53 ± 8.01	58.35 ± 12.77
Phe	43.32 ± 3.92^b^	50.42 ± 4.36^a^
Tyr	40.12 ± 6.96	43.73 ± 4.89
Trp	34.72 ± 1.53	34.8 ± 4.98
Glu	31.7 ± 3.90	34.67 ± 4.59
Tau	25.98 ± 11.01	25.42 ± 7.13
β-Ala	3.95 ± 1.62^B^	39.88 ± 22.65^A^
Asn	19.27 ± 4.82	23.08 ± 5.10
Met	19.2 ± 2.42	20.63 ± 4.07
Hyp	14.17 ± 2.55	15.67 ± 3.06
Hpro	15.73 ± 10.60	13.64 ± 3.05
3-MHis	6.80 ± 1.37	11.26 ± 4.89
Asp	7.86 ± 2.57	9.41 ± 3.33
Car	6.50 ± 2.72	8.73 ± 2.11
Abu	2.91 ± 0.47	2.88 ± 0.51
Kyn	2.20 ± 0.52	2.55 ± 0.33
Cys1	2.58 ± 1.11	2.00 ± 0.47
Ans	2.15 ± 1.15	1.67 ± 0.96
EtN	1.40 ± 0.70	1.48 ± 0.53
Harg	1.31 ± 0.43	1.37 ± 0.22
Aad	1.04 ± 0.42	1.11 ± 0.41
Sar	0.83 ± 0.24	0.96 ± 0.22
3-Aib	0.63 ± 0.20	1.00 ± 0.44
5-HT	0.66 ± 0.15^A^	0.38 ± 0.09^B^
Asa	0.62 ± 0.54	0.24 ± 0.28
Hcit	0.30 ± 0.19	0.28 ± 0.23
5-HTP	0.25 ± 0.10	0.29 ± 0.14
PEtN	0.53 ± 0.97	--
5-Ava	0.25 ± 0.32	0.18 ± 0.21
Cth	0.14 ± 0.12	0.22 ± 0.11
Hyl	0.10 ± 0.15	0.15 ± 0.29
GABA	0.05 ± 0.06	0.11 ± 0.14
Hser	0.10 ± 0.07	0.05 ± 0.08

^a,b^Values within a row without common superscripts differ significantly (*p* < 0.05). ^A,B^Values in rows with no common superscript, highly significant (*p* < 0.01).

As shown in Figure 3B and Table 5, post-competition plasma analysis displayed that the β-alanine concentration in the Yili horse test group was 6.12 times higher than that of the control group (*p* < 0.01). Moreover, concentrations of carnosine and 3-methylhistidine were significantly higher than those of the control group by 72.03 and 70.65% (*p* < 0.05), while valine concentrations were notably lower by 23.71% (*p* < 0.01). Notably, ethanolamine and taurine exhibited substantial changes in plasma concentrations before and after the race. Ethanolamine and taurine concentrations in the test group increased by 3.4 and 1.6 times before the race, respectively. Specifically, ethanolamine concentration in the test group remained higher than that of the control group before and after the race, whereas taurine concentration in the test group was lower than that of the control group immediately after the race. Regarding PCoA analysis, the horizontal coordinate contributed 50.53% to sample variance, while the vertical coordinate contributed 23.29%. Interestingly, there was high amino acid similarity observed in the plasma of control and test Yili horses immediately after the race, as revealed by the PCoA analysis. The PCA analysis reveals that the primary contributor to sample variance is the horizontal coordinate, amounting to 74.22%. Following this, the vertical coordinate represents the second principal component, contributing 19.68% to the sample variance. It exhibited a notable resemblance in the amino acid composition of plasma between control and test Yili horses immediately after the race.

**Table 5:** Effect of β-alanine on the metabolic concentration of amino acids in the plasma of Yili horses in the immediate post-race period (μmol/L).

Items	Control groups	Test groups
Gly	431.67 ± 254.72	412.33 ± 84.91
Ala	446.17 ± 92.74	358 ± 88.14
Gln	305.33 ± 43.27	308.5 ± 21.68
Val	255.17 ± 36.65^A^	194.67 ± 24.41^B^
Ser	177.33 ± 12.64	162.17 ± 22.09
Pro	112.07 ± 19.66	107.35 ± 10.61
Leu	113.33 ± 9.07	99.80 ± 17.63
Arg	80.70 ± 7.78	96.77 ± 18.39
Cit	76.33 ± 41.93	53.28 ± 5.83
Lys	90.9 ± 14.73	82.40 ± 18.00
Orn	61.93 ± 10.09	72.52 ± 15.70
Ile	68.52 ± 11.04	58.82 ± 8.56
His	53.67 ± 6.82	54.10 ± 10.43
Thr	61.73 ± 7.68	61.3 ± 10.94
Phe	48.5 ± 4.31	53.4 ± 5.22
Tyr	42.72 ± 4.69	47.83 ± 5.37
Trp	34.15 ± 3.27	29.9 ± 6.44
Glu	41.1 ± 6.53	46.03 ± 15.91
Tau	45.92 ± 14.79	40.5 ± 23.12
β-Ala	4.45 ± 0.81^B^	27.22 ± 13.51^A^
Asn	17.65 ± 7.54	17.02 ± 5.84
Met	18.78 ± 1.73	21.57 ± 5.19
Hyp	12.37 ± 2.17	13.43 ± 2.92
Hpro	10.46 ± 5.53	10.63 ± 2.37
3-MHis	7.60 ± 2.14^b^	12.97 ± 5.34^a^
Asp	10.86 ± 4.60	8.79 ± 4.71
Car	6.65 ± 2.96^B^	11.44 ± 1.22^A^
Abu	2.83 ± 0.88	2.45 ± 0.40
Kyn	2.15 ± 0.45	2.53 ± 0.15
Cys1	1.99 ± 1.73	0.78 ± 0.18
Ans	0.96 ± 0.6	0.83 ± 0.63
EtN	4.54 ± 3.18	5.00 ± 4.53
Harg	1.18 ± 0.4	0.76 ± 0.46
Aad	1.69 ± 2.00	0.76 ± 0.57
Sar	0.69 ± 0.26	0.98 ± 0.29
3-Aib	0.91 ± 0.26	0.84 ± 0.44
5-HT	1.63 ± 1.38	1.60 ± 2.01
Asa	0.18 ± 0.24	0.57 ± 0.41
Hcit	0.19 ± 0.25	0.35 ± 0.35
5-HTP	0.23 ± 0.18	0.36 ± 0.22
PEtN	2.21 ± 3.28	1.11 ± 1.24
5-Ava	0.35 ± 0.40	0.28 ± 0.20
Cth	0.30 ± 0.50	0.13 ± 0.12
Hyl	0.15 ± 0.11	0.09 ± 0.13
GABA	0.25 ± 0.24	0.07 ± 0.11
Hser	0.04 ± 0.06	0.05 ± 0.07

^a,b^Values within a row without common superscripts differ significantly (*p* < 0.05). ^A,B^Values in rows with no common superscript, highly significant (*p* < 0.01).

This similarity is evident in Figure 3C and Table 6, where the plasma concentrations of β-alanine, carnosine, and 3-methylhistidine in the Yili test group were significantly higher than those in the control group (*p* < 0.05) at the 2-h mark post-race. Conversely, the plasma concentrations of the major branched-chain amino acids (BCAAs)—valine, leucine, and isoleucine—were 31.88% (*p* < 0.01), 36.75% (*p* < 0.01), and 36.07% (*p* < 0.01) lower, respectively, in the Yili test group than in the control group at the same post-race interval. Plasma lysine levels changed immediately after the race, decreasing in both test and control groups, with the test group exhibiting significantly lower levels than the control group at the 2-h mark post-race (*p* < 0.05). The PCoA analysis results demonstrate that the horizontal coordinate accounts for 44.65% of sample variation, while the vertical coordinate represents other principal components contributing 23.38% to sample variation. There was a lack of significant amino acid similarity in the plasma of control and test Yili horses 2 h after racing. The PCA results showed that the horizontal coordinate is the main component, contributing 51.83% to the sample variance. The vertical indicates the second principal component, contributing 31.59% to the sample variance, showing a high similarity of amino acids in the plasma of horses in the control group and Yili horses of the test group 2 h after the race.

**Table 6:** Effect of β-alanine on the metabolic concentration of amino acids in the plasma of Yili horses 2 h after the race (μmol/L).

Items	Control groups	Test groups
Gly	316.00 ± 72.16	403.17 ± 99.20
Ala	371.67 ± 137.26	335.33 ± 113.53
Gln	283.67 ± 36.97	298.83 ± 49.09
Val	232.67 ± 23.85^A^	158.50 ± 15.31^B^
Ser	165.50 ± 24.44	148.00 ± 32.85
Pro	117.28 ± 42.71	118.10 ± 22.59
Leu	99.65 ± 15.01^A^	63.03 ± 9.94^B^
Arg	75.68 ± 14.87	79.50 ± 16.83
Cit	78.07 ± 35.72	66.48 ± 26.47
Lys	78.72 ± 16.69a	58.77 ± 10.97b
Orn	56.78 ± 12.45	50.62 ± 13.64
Ile	65.23 ± 11.36^A^	41.70 ± 6.28^B^
His	62.17 ± 11.05	56.40 ± 10.81
Thr	63.82 ± 7.22^a^	53.05 ± 7.57^b^
Phe	48.62 ± 6.07	49.53 ± 6.16
Tyr	43.95 ± 8.67	41.13 ± 6.02
Trp	39.08 ± 4.43	36.62 ± 4.25
Glu	32.38 ± 6.58	35.8 ± 13.17
Tau	23.66 ± 10.50	26.17 ± 12.96
β-Ala	4.13 ± 1.05^B^	27.47 ± 17.83^A^
Asn	22.90 ± 5.38	18.1 ± 5.00
Met	19.97 ± 2.80	21.78 ± 4.80
Hyp	15.73 ± 2.23	15.70 ± 3.89
Hpro	12.02 ± 7.67	10.76 ± 2.33
3MHis	8.43 ± 0.97^b^	13.52 ± 5.41^a^
Asp	6.93 ± 2.94	8.74 ± 4.59
Car	7.77 ± 2.06^b^	11.12 ± 2.79^a^
Abu	2.76 ± 0.70	2.38 ± 0.41
Kyn	2.09 ± 0.47^b^	2.62 ± 0.28^a^
Cys1	1.91 ± 1.24	1.50 ± 0.52
Ans	1.33 ± 0.37	1.59 ± 0.53
EtN	1.59 ± 0.90	1.68 ± 1.09
Harg	1.04 ± 0.19	1.01 ± 0.18
Aad	0.97 ± 0.26	1.00 ± 0.58
Sar	0.75 ± 0.12	0.8 ± 0.32
3-Aib	0.71 ± 0.15	0.90 ± 0.29
5-HT	0.8 ± 0.31	1.66 ± 2.46
Asa	0.25 ± 0.15	0.36 ± 0.46
Hcit	0.28 ± 0.17	0.3 ± 0.31
5-HTP	0.18 ± 0.19	0.06 ± 0.15
PEtN	0.27 ± 0.35	--
5-Ava	0.26 ± 0.22	0.29 ± 0.22
Cth	0.04 ± 0.10^b^	0.24 ± 0.16^a^
Hyl	0.20 ± 0.15	0.18 ± 0.19
GABA	0.24 ± 0.09	0.28 ± 0.11
Hser	0.02 ± 0.03	0.04 ± 0.07

^a,b^Values within a row without common superscripts differ significantly (*p* < 0.05). ^A,B^Values in rows with no common superscript, highly significant (*p* < 0.01).

### 3.4 Effect of β-alanine supplementation on blood gas indices before and after a 1,000 m speed race in Yili horses

**Figure 4:**
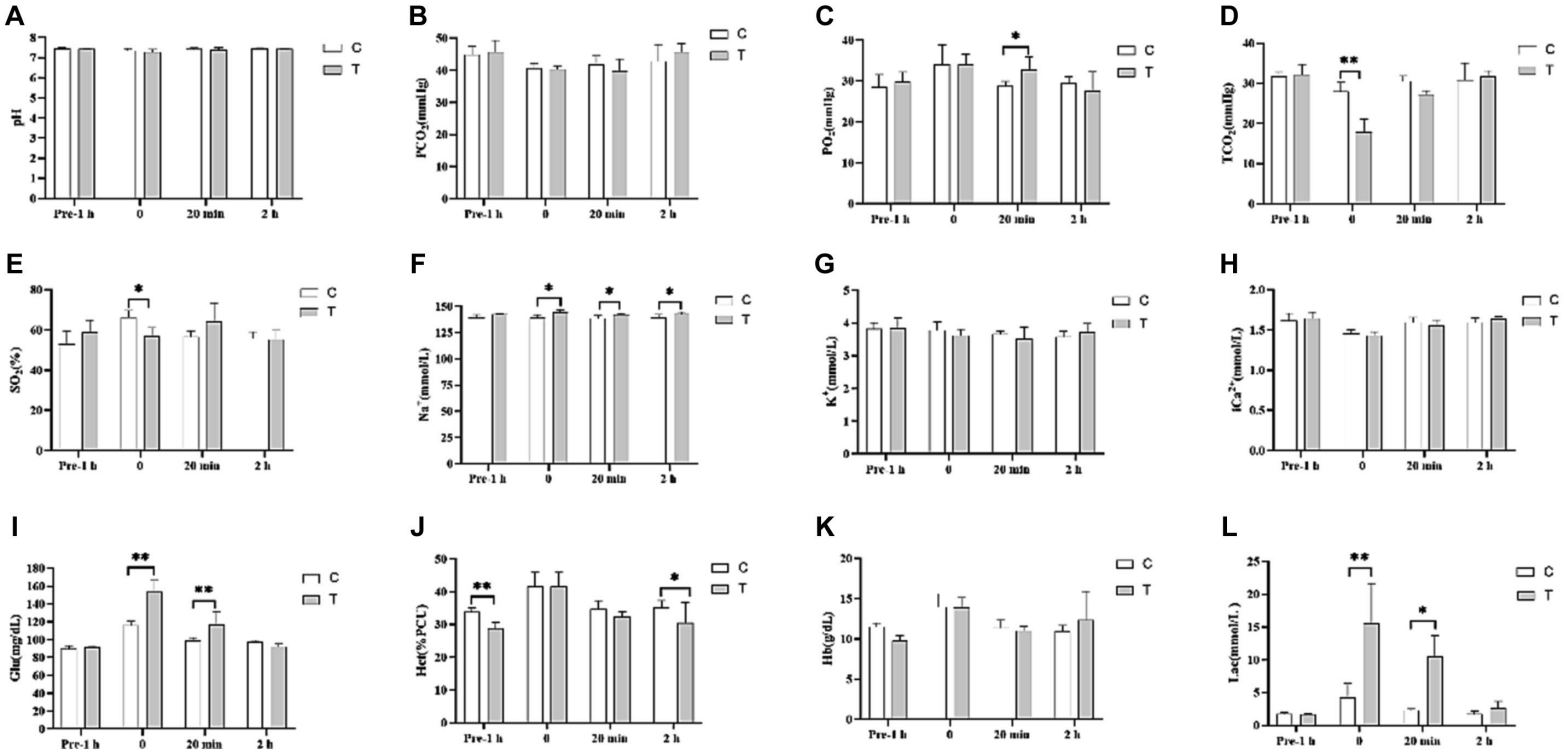
Effect of β-alanine supplementation on blood gas indices before and after a 1,000 m speed race in Yili horses (**A–L**) (C: α-alanine group T: β-alanine group). Each bar represents the mean of 6 horses ± standard error of the mean (SEM). * indicates a significant difference between the two groups (*p* < 0.05), ** indicates a highly significant difference between the two groups (*p* < 0.01).

**Figure 5:**
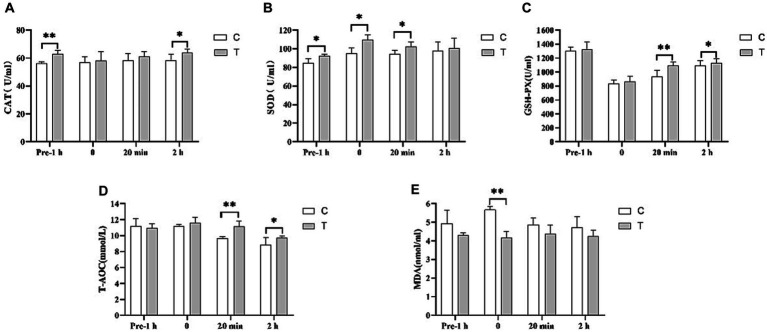
Effect of supplemental feeding of β-alanine on plasma antioxidant capacity before and after 1,000 m speed races in Yili horses. (**A–E**) Each bar represents the mean of 6 horses ± standard error of the mean (SEM). * indicates a significant difference between the two groups (*p* < 0.05), ** indicates a highly significant difference between the two groups (*p* < 0.01).

Figure 4 shows the effect of β-alanine on blood gas indices before and after the 1,000-m speed race in Yili horses. Notably, the VIP blood gas indexes of Glu and Lac in the control group were significantly lower than those in the test group by 24.58% (*p* < 0.01) and 72.48% (*p* < 0.01), respectively, in the immediate post-competition period. Among the non-VIP indicators, TCO_2_ and SO_2_ were 57.30% (*p* < 0.01) and 16.22% (*p* < 0.05) higher in the control group than in the test group, respectively. The differences in Glu and Lac levels between the two groups persisted at 20 min after the race, while PO_2_ was significantly higher in the test group than in the control group. Two hours after the race, the blood indexes had largely recovered, with the Hct of the control group remaining significantly higher than that of the test group. Figure 5 illustrates the effect of supplemental β-alanine feeding on antioxidant levels in Yili horses before and after a 1,000-m speed race.

### 3.5 Effect of supplemental feeding of β-alanine on plasma antioxidant capacity before and after 1,000 m speed races in Yili horses

In Figure 5A, the CAT activity in the plasma of the Yili horse test group was 11.70% higher than that of control group 1 h before the race (*p* < 0.01). There was no significant difference immediately after and 20 min after the race, but 2 h after the plasma CAT activity in the test group was notably higher than that of the control group 2 h after the race by 9.39% (*p* < 0.05). In Figure 5B, SOD activity in the plasma of Yili horses in the test group was 9.28%, 15.45%, and 8.66% higher than that of the control group 1 h before the race, immediately after, and 20 min after the race, respectively (*p* < 0.05). However, no significant difference was observed between the two groups 2 h after the race. Figure 5C displays no significant difference in GSH-PX activity in the plasma of Yili horses in the test group 1 h before and immediately after the race. Yet, 20 min and 2 h after the race, the test group showed significantly higher levels by 16.39% (*p* < 0.01) and 3.70% (*p* < 0.05), respectively, compared with the control group. In Figure 5D, T-AOC viability in the plasma of Yili horses in the test group showed no significant difference 1 h before and immediately after the race. However, it was significantly higher than that in the control group 20 min after (*p* < 0.01) and 2 h after the race (*p* < 0.05), by 15.40% (*p* < 0.01) and 9.84% (*p* < 0.05), respectively. Figure 5E indicates no significant difference in MDA activity in the plasma of Yili horses in the test group 1 h before, 20 min after, and 2 h after the race. Yet, immediately after the race, the test group exhibited a significant 26.46% reduction in MDA activity compared with the control group (*p* < 0.01).

### 3.6 Pearson’s correlation analysis of the top 10 amino acids in the plasma with blood gas indices and antioxidant capacity of 1,000 m speed-racing Yili horses supplemented with β-alanine before and after racing

**Figure 6:**
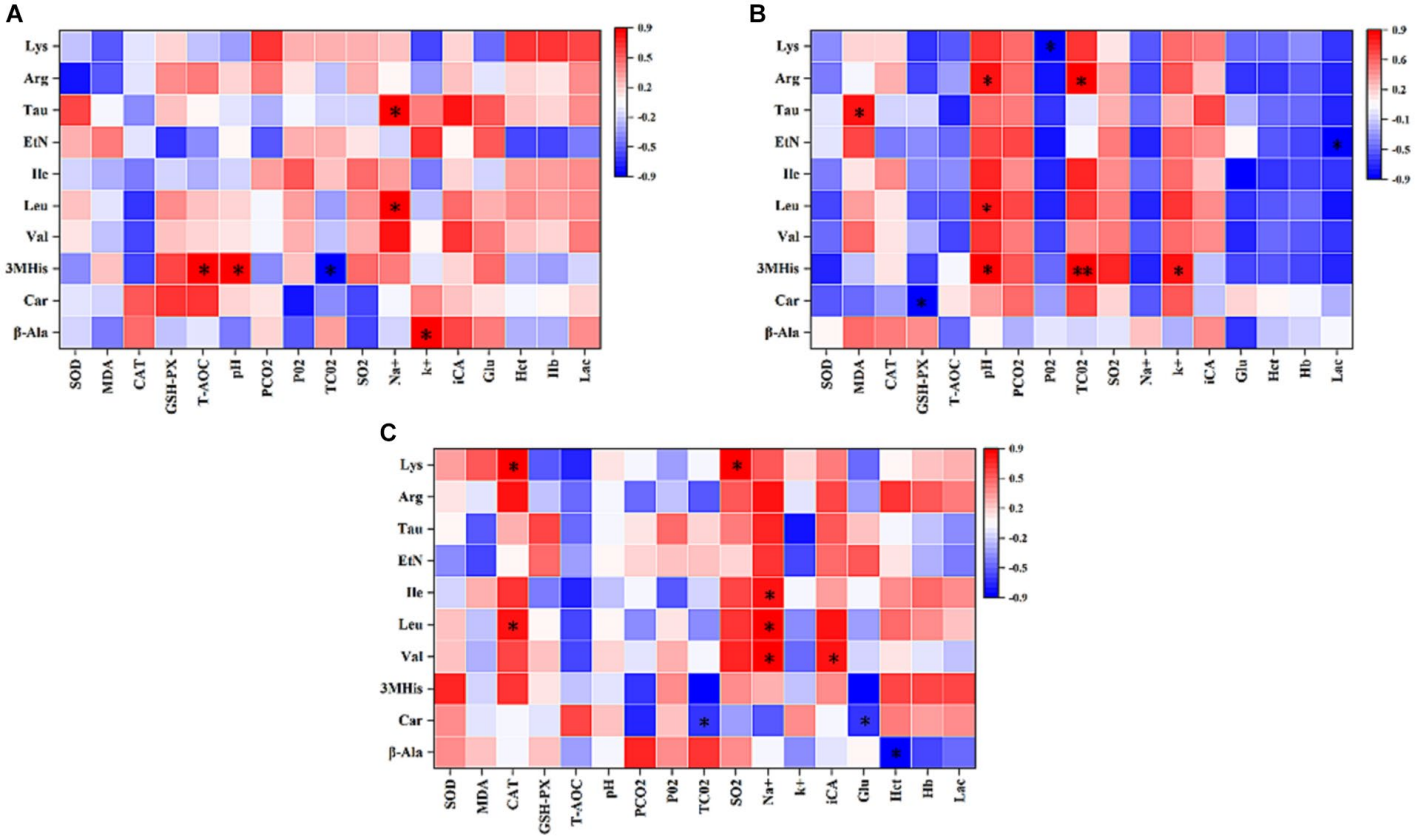
Pearson’s correlation analysis of the top 10 amino acids in the plasma with blood gas indices and antioxidant capacity of 1,000 m speed-racing Yili horses supplemented with β-alanine before and after racing (**A–C**) (C: α-alanine group T: β-alanine group). The intensity of the colors represents the degree of association. Red represents a significant positive correlation (*p* < 0.05), blue represents significantly negative correlation (*p* < 0.05), and white shows that the correlation was not significant (*p* > 0.05).

This study conducted an analysis of the Pearson’s correlation between the top 10 amino acids in plasma, blood gas indices, and antioxidant capacity before and after 1,000-m speed races involving Yili horses supplemented with β-alanine. Figure 6A demonstrates that 1 h before the race, T-AOC exhibited a significant positive correlation with 3MHis concerning antioxidant capacity. Moreover, pH was positively correlated with 3MHis, while TCO_2_ showed a negative correlation with 3MHis among the blood gas indexes. Additionally, Na^+^ exhibited a positive correlation with Leu and Tau, while K^+^ showed a positive correlation with β-alanine. Following the race, as shown in Figure 6B, there was a significant positive correlation in antioxidant capacity between MDA and Tau, and a significant negative correlation between GSH-PX and Car. Regarding blood gas indices, pH displayed a significant positive correlation with 3MHis, Leu, and Arg. Conversely, PO_2_ exhibited a significant negative correlation with Lys, while TCO_2_ showed either a significant or highly significant positive correlation with 3MHis and Arg. Moreover, K^+^ demonstrated a significant positive correlation with 3MHis, while Lac exhibited a significant negative correlation with Eth. The relationship between 3-MHis and β-alanine metabolism is direct, with 3MHis being a product of goose carnosine methylation. Elevated plasma levels of β-alanine in speed-racing Erythroxylon horses, subsequent to β-alanine supplementation, stimulate goose carnosine synthesis, leading to increased 3-methylhistidine. *In vivo*, 3-MHis exists in myofibrillar proteins and skeletal muscle myosin, releasing energy during myosin catabolism through oxidation. Notably, plasma 3MHis content displayed significant or highly significant positive correlation with blood gas indexes TCO_2_, K^+^, and pH immediately after the race. This suggests that Yili horses supplemented with β-alanine experienced heightened anaerobic respiration during the 1,000-m test race, resulting in increased lactate accumulation (Lac) and subsequently elevated blood gas indexes TCO_2_, K^+^, and pH. Figure 6C depicts that CAT in antioxidant capacity displayed a significant positive correlation with Leu and Lys at the 2-h mark after the race. Among the blood gas indices, TCO_2_ exhibited a significant negative correlation with Car, while SO_2_ showed a significant positive correlation with Lys. Furthermore, Na^+^ exhibited a significant positive correlation with Val, Leu, and Ile, whereas iCa displayed a significant positive correlation with Val. Glu, by contrast, demonstrated a significant negative correlation with Car, and Hct exhibited a significant negative correlation with β-alanine.

## 4 Discussion

Many studies have suggested the notable increase in skeletal muscle carnosine content through oral administration of β-alanine (12–14). Unlike direct carnosine administration, oral intake of β-alanine is readily absorbed by the intestines, entering the body’s circulation and reaching the myocytes to participate in carnosine synthesis within the myocytes (5, 16, 17). Dunnet’s study showed that supplemental with β-alanine 300 mg/kg BW/day to untrained pure blood horses gave the peak β-alanine concentration in the blood 6 h after the first dose (15). In our study, Yili horses supplemented with β-alanine showed a notably higher plasma β-alanine level compared with those supplemented with α-alanine 2 h, specifically at 9:00 post-supplementation. This level exhibited an upward trend during 15:00–18:00, gradually declining during 18:00–21:00. Unlike Dunnet’s study, our research observed a delayed peak and lacked a plateau (15). Dunnett previously established that blood carnosine predominantly originates from myocyte spillover after injury (15). However, in this study, no strenuous exercise occurred during the period of supplemental feeding, consequently resulting in no significant increase in plasma carnosine levels on the day of supplementation. Nevertheless, the present study conducted a follow-up assessment of β-alanine and carnosine levels in the blood of both groups on the 30th day of the trial. This analysis revealed a significant elevation in plasma β-alanine and carnosine levels among speed-raced Erythroxylon horses that were supplemented with β-alanine for 30 days.

Carnosine’s physicochemical properties render it beneficial for enhancing athletic performance. It can mitigate and buffer excessive acid production by reducing proteolytic metabolism, thereby contributing to muscle toning and strengthening (18–21). Muscle carnosine content plays a pivotal role in high-intensity and long-distance exercise (10). For instance, Wim et al. observed a performance improvement of 0.75 s among 400-m track and field athletes after a 4-week β-alanine supplementation compared with the control group (22). Similarly, Baguet et al. reported a 4.3-s improvement in race performance among 2,000 m professional rowers following a 7-week supplementation of 5 g/day β-alanine compared to the control group, which, prior to the trial, had a 0.3 s faster race time than the supplemented group (4). In our study, at the experiment’s outset, the 1,000-m exercise performance for both groups of speed racing Yili horses was recorded at 135.76 s and 134.73 s, respectively. After 30 days, the group of speed-racing Yili horses supplemented with β-alanine exhibited a decrease in their 1,000-m exercise performance by 13.29 s compared with the control group in terms of race time. This marked a significant performance improvement of 12.01%, consistent with the findings of Wim et al. and Baguet et al. This suggests that β-alanine supplementation can increase skeletal muscle carnosine content, mitigate physiological declines and energy depletion due to cellular acidification, and enhance the post-race recovery capability of speed-racing Yili horses, thereby improving their overall performance.

During exercise, the body undergoes changes in energy and substance metabolism, reflected in metabolite concentrations. Amino acids, integral to body metabolites, not only signify protein metabolism but also monitor pre- and post-exercise bodily changes. Studying amino acid variations pre- and post-exercise can offer insights into internal metabolism. In our research, speed-racing Yili horses supplemented with β-alanine exhibited notably higher plasma levels of β-alanine compared with those supplemented with α-alanine. Additionally, β-alanine supplementation resulted in significantly elevated plasma levels of carnosine in these horses. Dunnett et al. inferred that carnosine in plasma primarily stemmed from myocyte overflow post-injury (23). The plasma carnosine content in Yili horses supplemented with β-alanine notably increased immediately after and 2 h post-race. This suggests a substantial release of carnosine due to muscle injury during the race, indirectly supporting the notion that β-alanine supplementation aids carnosine synthesis in the skeletal muscle. Valine, leucine, and isoleucine, collectively known as BCAAs, are essential amino acids. BCAAs exhibit a rapid transamination and full oxidation capability, generating ATP at a higher rate compared to other amino acids (24). Consequently, they stand out as a crucial energy source during exercise, contributing to 14% of the total oxidative energy supply in skeletal muscle, with leucine displaying the highest activity in this regard (25). Blomstrand showed that increased utilization of BCAAs during high-intensity exercise leads to decreased plasma concentrations of BCAAs (26). In the present study, concentrations of leucine and isoleucine in the plasma of speed-racing horses were notably reduced both 1 h before and immediately after the race. This reduction was particularly significant in horses supplemented with β-alanine 2 h after the race, aligning with the results of Blomstrand’s study. This suggests that the utilization of BCAAs in the skeletal muscles of horses supplemented with β-alanine was higher than that of the speed-racing horses supplemented with α-alanine during the 1,000-m test event, resulting in substantial depletion of these amino acids. The utilization rate was notably higher in β-alanine supplemented speed-racing horses than in α-alanine supplemented. Thus, a large amount of BCAAs were consumed, resulting in lower plasma levels of BCAAs in β-alanine-supplemented speed racing horses 2 h after the race.

During moderate- to high-intensity exercise, the accumulation of hydrogen ions causes a decrease in intramuscular pH, influencing the muscle’s oxidative energy supply. Increased reliance on glycolysis for energy production leads to higher lactate (Lac) and hydrogen ion concentrations in the resulting products. Davery and Lily demonstrated that carnosine functions as an H^+^ buffer within the body’s metabolism, contributing to nearly 10% of the buffering capacity (24, 27). The hypothesis proposed by Blancquaert suggests that carnosine regulates the acid–base environment within myocytes by readily binding to Ca^2+^ and H^+^ within the cell, facilitating the transport of these ions inside the cell (10). During anaerobic respiration, myocytes within the sarcolemma metabolize glucose to generate ATP, resulting in a significant accumulation of H^+^ ions. These H^+^ ions competitively bind to the carnosine-Ca^2+^ complex, releasing Ca^2+^. The released Ca^2+^ near the sarcolemma immediately participates in muscle contraction. Subsequently, the carnosine-H^+^ complex moves toward the sarcoplasmic reticulum near the myocyte membrane. Simultaneously, it releases a substantial amount of H^+^ into the bloodstream to regulate the internal stability of myocytes. In our study, no significant differences in pH, TCO_2_, PCO_2_, Na^+^, K^+^, and iCa were observed between the two groups 1 h before the race. However, immediately after the race, β-alanine-supplemented speed-racing Yili horses exhibited notably higher blood levels of Glu, Lac, and Na^+^ compared to α-alanine-supplemented speed-racing Yili horses. Additionally, the pH was lower in the β-alanine-supplemented group. These differences could be attributed to their performance during the 1,000-m exercise. The anaerobic respiratory work performed by speed-racing Yili horses supplemented with β-alanine during the 1,000-m test race was greater than that of horses supplemented with α-alanine. Consequently, this led to increased production of Lac and H^+^, resulting in substantial Lac accumulation post-race. However, after two subsequent speed races, the Lac content in the whole blood of speed-racing Yili horses decreased from elevated levels to within the normal range. Additionally, other blood gas levels, such as pH, TCO_2_, PCO_2_, Na^+^, K^+^, and iCa, were restored to normal levels. This suggests that carnosine potentially enhances the myocytes’ acid–base environment by interacting with H^+^ and Ca^2+^ within the myocyte, leading to the normalization of Lac and pH levels. Hematocrit (Hct) demonstrates a positive correlation with blood viscosity during exercise, while blood viscosity shows a negative correlation with blood mobility. Higher blood viscosity adversely affects the perfusion of tissues and organs, leading to more pronounced tissue hypoxia. Conversely, lower blood viscosity enhances oxygen transport through the blood, augmenting oxygen supply to the tissues. This facilitates improved exercise levels and accelerates post-exercise recovery (28). In our study, pre- and post-race Hct levels, measured 2 h after racing, were notably lower in speed-racing Yili horses supplemented with β-alanine than in those supplemented with α-alanine. This suggests that β-alanine supplementation in speed-racing Yili horses enhances their resting blood oxygen supply capacity.

Several recent studies have illustrated carnosine’s extensive antioxidant properties, showcasing its ability to interact with various free radicals. Carnosine regulates the content of reactive oxygen species, effectively scavenges oxygen radicals, and serves as an antioxidant (2, 29). Additionally, carnosine directly interacts with peroxidation products of lipids and superoxide anion radicals, thereby improving the oxidative stability of skeletal muscle (30). High-intensity exercise generates substantial free radicals within skeletal muscle cells. Carnosine’s antioxidant mechanism involves neutralizing and binding to carbonyl groups on cell membrane proteins. This process produces relatively stable carbonylated proteins, preventing damaging cross-linking with other proteins and their expulsion from the cell. Guo’s research demonstrated that carnosine can effectively inhibit lipid oxidation in liver homogenates, reduce peroxidation-associated MDA, and decrease the accumulation of lipid oxidation products and then concluded that carnosine not only scavenges free radicals but also inhibits lipid peroxidation by reacting with lipid oxidation products and eliminating the accumulation of lipid oxidation products (31). Huang’s study revealed that administering β-alanine to SD rats for 4 weeks increased SOD and GSH-px activity in the gastrocnemius muscle in the high-dose group. Additionally, MDA content decreased significantly in the medium-dose group, substantially enhancing the skeletal muscle’s antioxidant capacity in SD rats (32). Wang’s findings showed that supplementing fattening pigs’ diet with 600 mg/kg β-alanine increased the activities of glutathione peroxidase and catalase while decreasing malondialdehyde concentration (33). In our study, plasma SOD activity in speed-racing horses supplemented with β-alanine was significantly higher than that of speed-racing horses supplemented with α-alanine, observed from 1 h before the race to 20 min after. GSH-px and T-AOC were notably higher in the β-alanine group at 20 min after the race, and MDA levels immediately post-race were significantly lower compared to horses supplemented with α-alanine. The enhanced antioxidant capacity in the plasma of speed-racing Yili horses corresponded to an increase in plasma taurine content. Taurine aids in neutralizing free radicals produced during exercise, minimizing oxidative stress damage to myocytes. This enables sustained energy supply to the myocytes and contributes to the improvement observed in the 1,000-m speed races of the β-alanine supplementation group (34).

## 5 Conclusion

In conclusion, supplementing with β-alanine enhances post-race acid–base recovery ability by elevating plasma levels of β-alanine and carnosine. This supplementation also improves the antioxidant status of speed-racing Yili horses, reducing post-exercise injuries and bolstering their post-race recovery ability. Consequently, it enhances the performance of Yili horses in 1,000-m races.

## Data availability statement

The original contributions presented in the study are included in the article/supplementary material, further inquiries can be directed to the corresponding author.

## Ethics statement

All procedures in this study were approved by the Animal Experiment Ethics Committee of Xinjiang Agricultural University (permit number: 2018012). The study was conducted in accordance with the local legislation and institutional requirements.

## Author contributions

XL: Writing – original draft. JM: Writing – original draft. HaiyL: Writing – review & editing. HaiL: Writing – review & editing. YM: Writing – original draft. HD: Writing – original draft. KY: Writing – review & editing.
